# Supplementary material for: Factors Associated with Combination Therapy Involving Traditional Korean Medicine in Pediatric Allergic Rhinitis Patients: A Retrospective Cohort Study Using National Health Insurance Data
Source: Healthcare (Basel). 2025 Apr 11;13(8):875. doi: 10.3390/healthcare13080875 (PMC12026663; doi:10.3390/healthcare13080875)
Supplement: Supplementary file 1 [file healthcare-13-00875-s001.zip › healthcare-3491268-supplementary.pdf]

Supplementary Table S1. The list of immunological tests and second-generation antihistamines

**Immunological tests**	Immunoglobulin-Ig E (D741003)<br>Total Ig E (D7429, D7430, D7431)<br>Allergen Specific Immunoglobulin-Ig E (D744001)<br>Allergen Specific Ig E Test by Kit (D7450)<br>Allergen Specific Ig E (D7451)<br>Allergen-Specific Ig E by MAST(D7460)<br>Histamine, immunoassay (D7480, D7481)<br>Nasal Provocation Test (Non-Specific) (E7124)<br>Nasal Provocation Test (Specific) (E7125)<br>Allergen Skin Test (Skin Prick Test) (E7151)<br>Allergen Skin Test (Intradermal Test) (E7152)<br>Skin Test (Allergen Test, Skin Prick Test) (EY853)<br>Skin Test (Allergen Test, Intradermal Test) (EY854)
**Antihistamines**	Acrivastine, Azelastine, Azelastine hydrogen chloride<br>Bepotastine, Cetirizine, Ebastine, Epinastine, Loratadine<br>Mizolastine, Olopatadine hydrochloride, Rupatadine

Supplementary Table S2. The list of diseases and diagnostic codes used for subject extraction

Diagnosis	KCD-7 List Used in Data Extraction	Comments
Allergic rhinitis	J30, J30.1, J30.2, J30.3, J30.4	Enroll criteria
Atopic dermatitis	L20, L20.88, L20.8, L20.9	Enroll criteria, Covariable
Asthma	J45*	Enroll criteria, Covariable
Allergic conjunctivitis	H10*	Enroll criteria, Covariable
Sinusitis	J01, J32	Covariable
Pneumonia	J13, J14, J15, J16, J17, J18	Covariable
Cancer	C*, D*	Exclusion criteria
Intracranial hemorrhage	I60-I62	Exclusion criteria
Cerebral infarction	I63	Exclusion criteria
Stroke,	I64	Exclusion criteria
Other cerebrovascular diseases	I65-I69	Exclusion criteria
Renal failure	N17-N19	Exclusion criteria

J30; vasomotor and allergic rhinitis (includes: spasmodic rhinorrhea, excludes: allergic rhinitis with asthma), J30.1; allergic rhinitis due to pollen, J30.2; other seasonal sllergic rhinitis, J30.3; other allergic rhinitis, J30.4; allergic thinitis, unspecified.

L20; atopic dematitis, L20.88; other atopic dermatitis, L20.8; other atopic dematitis, L20.9; atopic dematitis, unspecified.

J45*; all kind of asthma

H10*; all kind of conjuctivitis

J01; acute sinusitis, J32; chronic sinusitis

J13; pneumonia due to streptococcus pneumoniae, J14; pneumonia due to haemophilus influenzae, J15; bacterial pneumonia, nec (includes: bronchopneumonia due to bacteria other than S. pneumoniae and H. influenzae, excludes: Chlamydial pneumonia), J16; pneumonia due to other infectious organisms, nec (excludes: ornithosis, pneumocytosis, peumonia NOS, congenital pneumonia), J17; pneumonia in diseases classified elsewhere, J18; pneumonia, organism unspecified.

C*, D*; neoplasms

I60; subarachnoid haemorrhage, I61; intracerebral haemorrhage, I62; other nontraumatic intracranial haemorrhage

I63; cerebral infarction

I64; stroke, not specified as haemorrhage or infarction

I65; occlusion and stenosis of precerebral arteries, not resulting in cerebral infarction, I66; occlusion and stenosis of cerebral arteries, not resulting in cerebral infarction, I67; other cerebrovascular diseases, I68; cerebrovascular disorders in diseases classified elsewhere, I69; sequelae of cerebrovascular disease
N17; acute renal failure, N18; chronic kidney disease, N19; unspecified kidney failure
